# Effect of Combined High Pressure and Thermal Treatment on Myofibrillar Proteins Solubilization of Beef Muscle

**DOI:** 10.3390/ijms12053034

**Published:** 2011-05-11

**Authors:** Hanjun Ma, Guanghong Zhou, David A. Ledward, Xiaoling Yu, Runshu Pan

**Affiliations:** 1 School of Food Science, Henan Institute of Science and Technology, Xinxiang 453003, China; E-Mails: yuxiaoling@163.com (X.Y.); prsh@hist.edu.cn (R.P.); 2 Key Laboratory of Food Processing and Quality Control, College of Food Science and Technology, Nanjing Agricultural University, Nanjing 210095, China; E-Mail: ghzhou@njau.edu.cn; 3 Department of Food Biosciences, University of Reading, Whiteknights, Reading RG66AP, UK; E-Mail: david.ledward@nottingham.ac.uk

**Keywords:** high pressure, keyword, myofibril, protein solubilization, electrophoresis

## Abstract

The effects of high pressure (to 600 MPa) at different temperatures (20 to 60 °C) for 20 min on protein solubilization and electrophoretic pattern in beef post-rigor longissimus dorsi muscle were studied. The results showed that protein solubilization increased with increasing temperature, especially from 40 °C to 60 °C. A regular trend of protein solubilization was found when isolated myofibrils were subjected to high pressure at different temperatures, an increase was observed with increasing pressure up to about 400 MPa, solubility then decreasing to 600 MPa. Electrophoretic profiles showed that myosin light chains and actin thin filaments were sensitive to pressure, and were released from myofibrils subjected to 100 MPa and higher pressures at the different temperatures.

## 1. Introduction

High pressure processing is becoming increasingly used by the meat industry, as it can extend shelf life and improve the eating quality and functional properties of meat and meat products [1–3]. High pressure treatment can tenderize meat when applied pre-rigor, but it does not necessarily have such an effect on post-rigor meat at room temperature (20 °C) [4]. However, it is difficult for the industry to treat pre-rigor meat with high pressure [5]. Spores at ambient temperature can resist pressures up to 1000 MPa, but lower pressures (250 MPa) associated with mild temperatures (40 °C) can inactivate spores in a two stage process, pressure first inducing germination and then inactivating the baro-sensitive germinated spores [6], showing the benefits of combining high pressure technology with heat treatment. Therefore, high pressure technology can be applied in combination with heat treatment.

Myofibrillar proteins have a significant relationship with meat functional properties, therefore it is important to understand the changes that happen to the myofibrillar proteins. Many previous investigations have shown that as a consequence of depolymerization, pressure induces increased solubilization of myofibrillar proteins. Macfarlane *et al.* reported that when ovine meat is pressurized at 150 MPa, a marked increase in the yield of solubilized myofibrillar proteins occurs, but the effects were dependent on pH, temperature, and salt type and concentration [7,8]. Similar observations were observed by Suzuki *et al.* in rabbit meat [9]. These authors found that proteins from the thin filament such as actin, tropomyosin, troponin C as well as M-protein were solubilized at 100 MPa, whereas solubilization of myosin heavy chains required higher pressures (300 MPa). McArthur and Wilding [10] observed no solubilization of myosin heavy chains, even at 500 MPa, although myosin was shown to be partly denatured by differential scanning calorimetry.

The changes in myofibrillar proteins subjected to high pressures have been studied, but these studies were limited to ambient temperature. In the present study the effects of combined heat and pressure treatments on isolated myofibril solubility and protein electrophoretic pattern were investigated. The objective was to further understand the relative effects of heat and pressure treatments on the myofibrillar proteins of beef muscle.

## 2. Results and Discussion

### 2.1. Effect of Pressure and Heat on the Solubilization of the Proteins

**Figure 1.**
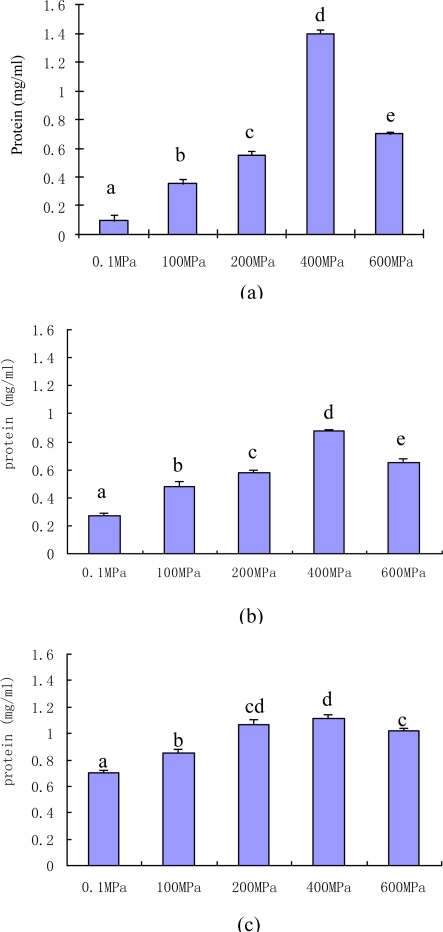
Changes in the concentration of myofibrillar protein in the supernatants obtained on centrifugation of the myofribillar extract after pressure treatment at **(a)** 20 °C, **(b)** 40 °C, and **(c)** 60 °C. 0.1 MPa is ambient pressure (control); a–e: means with different letters are significantly different (P < 0.05) between pressure treatments at a given processing temperature.

The effects of high pressure and temperature on the solubilization of myofibrillar proteins are presented in Figure 1.

On heat treatment at ambient pressure, the expected increase in the solubility of myofibrillar proteins with increasing temperature (P < 0.05), especially from 40 °C to 60 °C, was observed, where the concentration of soluble myofibrillar proteins increased from 0.273 mg/mL to 0.747 mg/mL.

At 20 °C, increasing pressure led to an increase in solubility of myofibrillar proteins, up to 400 MPa (1.403 mg/mL), the highest concentration for all treatments, after which a significant decrease with a further increase in pressure was observed. The pressure induced solubilization of beef myofibrillar proteins is similar to the results obtained for chicken [11]. Jung, Lamballerie-Anton and Ghoul also found increases in soluble protein at 300 MPa compared to the control and treatment at 100 MPa, but there was no significant change when pressure was increased from 300 to 600 MPa [12].

In comparison with the results obtained at 20 °C similar trends of protein solubilization were found when isolated myofibrils were subjected to pressure at 40 °C and 60 °C, an increase being observed with the increasing pressure up to 400 MPa, with a subsequent decrease at 600 MPa. Although similar trends of protein solubilization were found for pressure treatment at all temperatures, the extent and variability of the concentrations of soluble myofibrillar proteins differed at related pressures, which may be due to interactions between pressure and temperature.

### 2.2. Effect of Combined Pressure and Thermal Treatment on the Myofibrillar Proteins by SDS-PAGE Analysis

**Figure 2.**
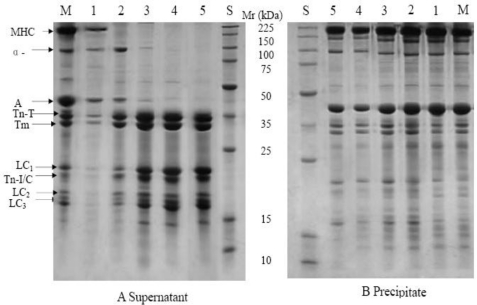
SDS-PAGE profiles from supernatants (**A**) and precipitates (**B**) of myofibrils after pressure treatment at 20 °C. M: myofibril; S: molecular weight standards; 1, 2, 3, 4 and 5 represent control samples and samples after treatment at 100, 200, 400, 600 MPa, respectively. MHC: myosin heavy chain; α: α-actinin; A: actin; Tn-T: troponin T; Tm: tropomyosin; Tn-I: troponin I; Tn-C: troponin C; LC1, 2, 3 represent myosin light chains 1, 2, 3, respectively.

SDS-PAGE profiles from supernatants and precipitates of the myofibrils after pressure treatment at 20 °C are shown in Figure 2. From Figure 2 and the relative intensity of the bands (data not shown), it was found that proteins molecular weights over 41 kDa decreased in the supernatant, while proteins in the molecular weight range 35–16 kDa increased in both the supernatant and precipitate when samples were subjected to pressures over 100 MPa, due to depolymerization of the myofibrillar proteins treated at higher pressures.

Myosin heavy chain was observed in the supernatant from the control but was absent in the pressure treated samples. This is in agreement with Yamamoto *et al.* who found that the band of the myosin heavy chain was not observed in samples pressurized at 300 MPa for 10 to 30 min [11]. This was attributed to the fact that the myosin heavy chain is not stable to pressurization and denatures to appear in the precipitate. The solubility of the myosin light chain increased with increasing pressure, as demonstrated by changes in the density of this band in the supernatant profiles.

α-Actinin is a component of the Z line of the myofibril. After treatment at 100 MPa, the density of this band in the supernatant was greater than in the control, but it disappeared gradually with increasing pressure, indicating that the solubility of α-actinin increased when subjected to 100 MPa at room temperature but decreased thereafter. Actin was observed in the supernatant of samples pressurized at 100 MPa for 20 min, but disappeared at pressures above 200 MPa. This result differs from Yamamoto *et al.* who observed actin in the supernatants after treatment at 300 and 400 MPa that was unchanged with increasing pressure [11]. Troponin T, I/C and Tropomyosin, which are major components of thin filaments, were solubilized by pressure treatment, as the densities of these protein bands increased in the supernatants and decreased in the precipitates with increasing pressure. Thin filaments are sensitive to pressure and depolymerize into G-actin [13]. June *et al.* also reported that the solubility of Troponin T, Tropomyosin and myosin light chains increased after pressure treatment [12].

**Figure 3.**
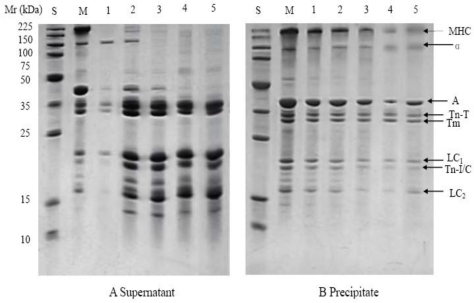
SDS-PAGE profiles from supernatants (**A**) and precipitates (**B**) of myofibrils after pressure treatment at 40 °C. M: myofibril; S: molecular weight standards; 1, 2, 3, 4 and 5 represent control samples and samples after treatment at 100, 200, 400, 600 MPa, respectively. MHC: myosin heavy chain; α: α-actinin; A: actin; Tn-T: troponin T; Tm: tropomyosin; Tn-I: troponin I; Tn-C:troponin C; LC1, 3 represent myosin light chains 1 and 3, respectively.

**Figure 4.**
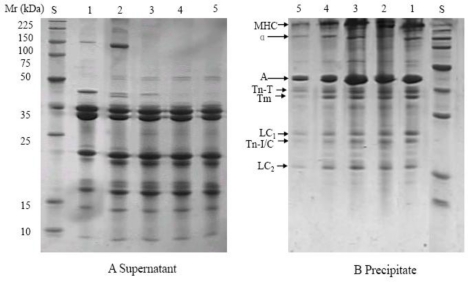
SDS-PAGE profiles from the supernatants (**A**) and precipitates (**B**) of myofibril after pressure treatment at 60 °C. S: molecular weight standards; 1, 2, 3, 4 and 5 represent control samples and samples after treatment at 100, 200, 400, 600MPa. MHC: myosin heavy chain; α: α-actinin; A: actin; Tn-T: troponin T; Tm: tropomyosin; Tn-I: troponin I; Tn-C:troponin C; LC1, 3 represent myosin light chains 1 and 3, respectively.

In the samples pressure treated at 40 °C and 60 °C, similar protein solubility changes were found (Figure 3 and Figure 4). New banding patterns were observed. Lower molecular weight bands (below 35 KDa) increased with the pressure up to 200 or 400 MPa in the supernatants, but there were no significant changes in the precipitates. These results are similar to those of Sikes *et al.* who found that when the myofibrillar proteins of beef neck were treated at 200 MPa and 60 °C for 20 min a new type of structure between myosin and actin was formed and the myofibrillar proteins degraded to smaller molecules [14]. In the present study the band with the molecular weight of about 18 KDa (LC2) disappeared in the samples pressure treated at 40 °C and 60 °C, presumably due to the interactive effect of pressure and temperature treatment.

## 3. Experimental Section

### 3.1. Sample Preparation

The beef Longissimus dorsi was obtained from a supermarket in Nanjing, China. The meat was from two ∼20–24 month old Luxi × Limousin crossbreeds, the weight of each side of the carcass was about 130 kg and had been kept at 4 °C for 3 days following slaughter. The beef sample was trimmed of all visible fat and cut into approximately 3 × 3 × 6 cm pieces with the fibers parallel to the longest axis, and packed in Multivac bags (Bosley International, London, UK), which were maintained at 4 °C for over 48 h until required [15].

### 3.2. Myofibrillar Extraction

Myofibrils were extracted using the method of Busch *et al.* [16]. Minced beef was homogenized for 20 s in a Waring blender (Stomacher 400, Paris, France) with six volumes of extraction buffer (20 mM Tris–HCl, pH 7.6, 5 mM EDTA). After centifugation at 1000 g for 10 min at 4 °C, the pellets were resuspended in the extraction buffer and the operation repeated five times. After the last centrifugation, the pellets were resuspended in five volumes of extraction buffer and homogenized in a Waring blender for 25 s. In order to remove the connective tissue, the homogenate was filtered through a 20 mesh nylon net (Shanghai Hailiang Filter Cloth CO., Ltd., Shanghai, China), centrifuged at 1000 g for 10 min and washed with the buffer. The pellets were resuspended in 100 mM KCl, centrifuged under the same conditions and finally homogenized in 100 mM KCl. The concentration of the solution was adjusted to 6 mg/mL. Samples of 10 mL were sealed in Multivac bags (Bosley International, London, UK) for treatment.

### 3.3. High Pressure and Heat Treatment

Myofibrils were treated at 100 to 600 MPa at room temperature, 40 and 60 °C for 20 min in the high pressure rig (Kefa New Technology Food Machine, Ltd., Baotou, China). The pressure unit comprised a 3 L cylindrical pressure chamber fitted with a thermoregulated system. Room temperature was the control, 20 °C. Some myofibrillar samples were heated, in water baths at 40 and 60 °C for 20 min, as controls. All experiments were carried out in triplicate.

### 3.4. Assay of Protein Solubility

After pressure treatment, samples of myofibrils were centrifuged for 20 min at 14,000 g and 4 °C, supernatants and precipitates from each sample were collected. The protein concentration of the supernatant was determined by BCA kit (Pierce Biotechnology, Int., Rockford, IL, USA).

### 3.5. Electrophoretic Analysis

The SDS–PAGE analysis was conducted using a 12.5% acrylamide gradient separating gel and 4% acrylamide stacking gel, as in Laemmli (1970) [16]. Samples of supernatant and precipitate (mixed with 8 volumes of buffer) were mixed with SDS-PAGE sample buffer (4% SDS, 20% glycerol, 0.125 M Tris–HCl, 10% β-mercaptoethanol [pH 6.87]) in a 1:1 (v/v) ratio were heated in boiling water for 3 min. Aliquots of 20 μg of protein per lane were loaded onto the acrylamide gel. Electrophoresis was first run at 80 V for about 30 min, and then at 120 V for 3 h. Gel was stained in Coomassie brilliant blue R-250 (0.1% Coomassie brilliant blue R-250, 45% methanol, 10% acetic acid) for 2 h. Destaining was in 10% methanol, 10% acetic acid for 12 h.

### 3.6. Statistical Analysis

Data were analyzed by a two way analysis of variance. A confidence level of 5% was used to compare means (P < 0.05). When significance was detected between samples, the mean values were compared using Fisher’s least significant difference (LSD) procedure.

## 4. Conclusions

The solubility of myofibrillar proteins varied with pressure and temperature, but the highest solubility was induced by treatment at room temperature and 400 MPa. Myosin light chains and actin thin filaments were sensitive to pressure, and were released from myofibrils subjected to 100 MPa and higher pressures at the different temperatures tested.
